# M. Larrey on Aneurism of the Heart

**Published:** 1842-04-30

**Authors:** 


*Aneurism of the Heart.*—At the Academy of Sciences, Paris, M. Larrey read a memoir on aneurism of the heart. In a former paper the author had endeavoured to prove that this disease is not incurable, if treated before it has arrived at the last period. The means employed by M. Larrey are, the application of cupping glasses over the left hypochondrium and back, followed by moxas; general bleeding, the use of ice, especially at the commencement, and antispasmodics. The moxa, according to M. Larrey, possesses an exciting property, the effects of which extend to the heart, and tend to restore the weakened parietes of the heart to their natural condition. The application of ice, curiously enough, acts in the same way. M. Larrey presented the cases of four women, treated by him on the above mentioned principles. In one case, fifty-nine moxæ and one hundred applications of the cupping glass had been resorted to.—*Provincial Medical and Surgical Journal.* March 5th, 1842.

